# Minimus: a fast, lightweight genome assembler

**DOI:** 10.1186/1471-2105-8-64

**Published:** 2007-02-26

**Authors:** Daniel D Sommer, Arthur L Delcher, Steven L Salzberg, Mihai Pop

**Affiliations:** 1 Center for Bioinformatics and Computational Biology, University of Maryland, College Park, MD 20742, USA; 2 Computer Science Department, University of Maryland, College Park, MD 20742, USA

## Abstract

**Background:**

Genome assemblers have grown very large and complex in response to the need for algorithms to handle the challenges of large whole-genome sequencing projects. Many of the most common uses of assemblers, however, are best served by a simpler type of assembler that requires fewer software components, uses less memory, and is far easier to install and run.

**Results:**

We have developed the Minimus assembler to address these issues, and tested it on a range of assembly problems. We show that Minimus performs well on several small assembly tasks, including the assembly of viral genomes, individual genes, and BAC clones. In addition, we evaluate Minimus' performance in assembling bacterial genomes in order to assess its suitability as a component of a larger assembly pipeline. We show that, unlike other software currently used for these tasks, Minimus produces significantly fewer assembly errors, at the cost of generating a more fragmented assembly.

**Conclusion:**

We find that for small genomes and other small assembly tasks, Minimus is faster and far more flexible than existing tools. Due to its small size and modular design Minimus is perfectly suited to be a component of complex assembly pipelines. Minimus is released as an open-source software project and the code is available as part of the AMOS project at Sourceforge.

## Background

With the advent of whole-genome shotgun (WGS) sequencing in the mid-1990s, the genomics community had an urgent need for software that could process tens of thousands of individual sequence "reads" and assemble those into the genome from which they had come. The first generation of assemblers, including TIGR Assembler [1], phrap [2], and CAP3 [3], were able to assemble small- to medium-sized bacterial genomes, often requiring several weeks of computer time on the fastest computers then available. As sequencing technology improved, ever larger projects were attempted with the WGS method, and it became clear that new methods were needed. For the 130 million base pair (Mbp) genome of the fruit fly *Drosophila melanogaster*, an entirely new assembler was developed [4], which incorporated many new ideas about efficient memory usage and sophisticated repeat processing. The Celera Assembler (CelAsm) was also the first algorithm to use mate pair information to any serious degree: taking advantage of the fact that most reads in a WGS project are generated in pairs, that system used the expected distance between reads in a pair to impose many useful constraints on the overall assembly. Other large-scale WGS assemblers followed, including Arachne [5,6], which was used to assemble the 2.6 billion base pair (Gbp) mouse genome [7], Phusion [8], Atlas [9], and JAZZ [10].

As these systems have scaled up to meet the needs of very large WGS projects, they have grown in size and complexity, so that today, only a few very sophisticated bioinformatics groups have the expertise needed to install and run them. Like many large systems, these assemblers are relatively brittle, meaning that they often crash if the data does not conform to fairly rigid specifications. However, because they produce far superior results to the first generation of assemblers, the leading genome centers have focused their efforts on these large assemblers to the exclusion of other approaches.

Meanwhile, a host of new genome sequencing applications has arisen that place different demands on assembly algorithms. Although large-scale sequencing has pushed assembly technology in productive directions, small-scale sequencing efforts have proliferated as well. Our group recognized the growing need for an assembler that could assemble a handful of sequencing reads with a minimum of overhead (both computational and human), and as a result we have developed *Minimus*, a fast, "lightweight" assembler that addresses these needs. Before describing the algorithm and our results, we will describe several of these motivating applications.

### Gap closure

Since the very first bacterial genome, *Haemophilus influenza *[11], was sequenced, we and our colleagues at The Institute for Genomic Research (TIGR) have been developing methods for closing the gaps in a draft genome. The initial assembly of a WGS project normally produces a large collection of contiguous pieces of DNA (contigs) that are separated by gaps. Improvements in sequencing and assembly technology have yielded fewer gaps per megabase in recent years, but nonetheless, the increased scale of sequencing has meant that large centers have many more gaps to fill. One unintended by-product of this trend is that many genomes today are left in "draft" form: the initial assembly is the only assembly, and the published genome consists of hundreds or thousands of unordered contigs.

Fortunately, many genomes, especially those of the greatest scientific interest, are still being finished, which means that all gaps need to be closed. Gap closure consists of running additional sequencing reactions that fill in the gap between two adjacent contigs. If the gap is filled with repetitive sequence (which is often the case), then "closure" teams may go to great lengths to clone and sequence small DNA fragments that correctly span the gap. Once these sequences are generated, the final step is to assemble the gap. This requires that the newly generated sequences, often spanning just a few kilobases or even less, be assembled together with the two surrounding contigs.

Large-scale assemblers such as CelAsm and Arachne can be used for this task, but this presents several problems. First, the scale of these programs means that simply loading them into memory can take longer than the execution time of the assembly itself. Second, the laboratory teams filling gaps typically use graphical tools to manage gaps, and configuring these tools to call a very large external program is impractical if not impossible. Third, and perhaps most telling, the cleverness of these WGS assemblers is a hindrance for gap closure, because the data do not conform to the characteristics of a typical shotgun process. The depth of coverage of finishing reads often differs from that in the surrounding areas thereby confusing the statistical repeat detection mechanisms present in large-scale assemblers, and preventing a correct assembly of the gap. Therefore an assembler for gaps will do better by using a simple, straight-forward algorithm, focused on a specific region of the genome. Finally, these assemblers cannot be easily modified to address the specific issues raised by specialized finishing procedures, especially as new finishing techniques are continuously being developed. For example, high-throughput finishing experiments often use transposons to sample a problematic region, resulting in paired reads that are facing away from each other (the sequencing proceeds away from the transposon). Such constraints cannot be easily incorporated in existing assemblers which are hard-coded to assume paired reads are facing inwards, towards the middle of the corresponding shotgun fragment. Flexible tools like Minimus and AMOS provide the potential for incorporating such information through add-on modules.

### Gene assembly

Another important use of small-scale assembly takes advantage of the rapidly growing Trace Archive at NCBI [12], a public repository of all the raw data from many large sequencing projects. Because it takes months and sometimes years before the final, assembled sequence from a genome project is released, scientists use the BLAST search function at the Trace Archive to find reads matching a gene of interest. If the gene is contained in the Trace Archive data, then a search will return anywhere from a handful to a few hundred sequences. These need to be assembled together to produce a better picture of the genomic region containing the gene. Once again, the scientist needs a small, less finicky assembler for this purpose.

### Small genomes

Although most sequencing capacity is taken up by the largest genome projects, the number of small genomes being sequenced easily outstrips – in number of species and strains – the number of large genomes. Ironically, some of the very clever and complicated ideas that make CelAsm, Arachne, and other assemblers work for large genomes make them less than ideal for these small genomes. Viruses are a good example: they typically have genomes ranging from 5–50 kilobases, and they contain relatively little repetitive DNA. Thus there is no need to characterize the repeat content, and a simple assembler that ignores the issues of large-scale WGS projects will produce a perfectly correct assembly more quickly. For example, the Influenza Genome Sequencing Project, which uses an RT-PCR strategy rather than WGS, has assembled over 1000 influenza genomes using Minimus [13], with savings coming from not having to address special formatting requirements to prepare the data and from not having to maintain a large assembly software package.

## Implementation

### Implementation details of Minimus

The Minimus assembler was built in a modular fashion from software modules available within the AMOS assembly package [25] and is released as one of the components of this package. AMOS is an open-source software package that provides researchers with a collection of modules and software libraries that are useful in the development of genome assembly and analysis software. A full description of the AMOS package is beyond the scope of this paper and will be published elsewhere (M.Pop, manuscript in preparation).

**Figure 1:**
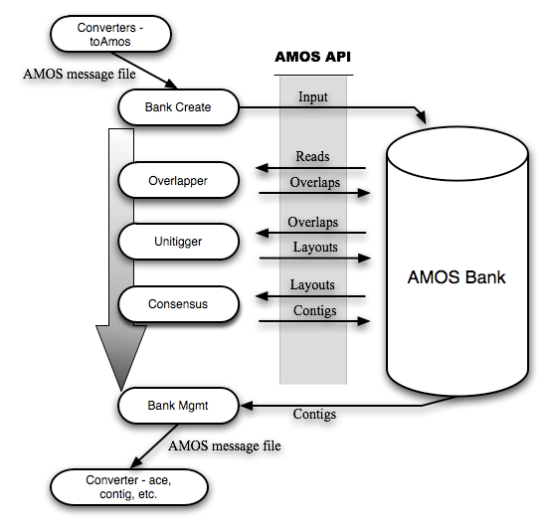
Overview of Minimus pipeline. Several independent modules of the AMOS package (shown as ovals) interact through the AMOS API to a central data-structure (called a Bank). The order of execution of the individual modules is shown by the arrow. Note that the inputs and outputs to minimus follow the AMOS file format (AMOS message files). The AMOS package provides converters between this file format and virtually all commonly used formats for representing sequence data and genome assemblies.

Minimus consists of the combination of three AMOS modules, following the traditional overlap-layout-consensus paradigm [26]. These modules interact with each other through a central AMOS data-structure (called a **bank**) as shown in Figure 1. The three modules are:

1. **hash-overlap **– a sequence overlapper that uses minimizers [27] to increase speed and decrease memory usage.

2. **tigger **– a unitigger, i.e. tool that identifies clusters of reads which can be uniquely assembled based on algorithms developed by Myers [28,29]; in graph theoretic terms a unitigger identifies maximal interval subgraphs of the overlap graph.

3. **make-consensus **– a progressive multiple alignment program that refines the read layout generated by the unitigger to build a precise multiple alignment of these reads.

Note that sequence quality values are only used during the generation of the multiple alignment consensus (step 3). Other assemblers, such as phrap, use the quality values as an integral component of the assembly algorithm. We found that, due to the high quality of data produced by modern sequencing instruments, the explicit consideration of quality values during the overlap and unitigging steps is unnecessary. Instead we only use the quality data to trim the poor quality flanks of each read (see below under Sequence trimming), and to compute the consensus (and associated quality values) for the multiple alignment of co-assembled reads.

An execution of Minimus consists of the following stages, described in detail below.

#### Input stage

The shotgun reads are loaded into the AMOS bank. The inputs are presented as an AMOS message file, whose format is modeled on the format used by Celera Assembler [4]. Virtually any existing format for representing shotgun data can be easily converted to this message format with the help of conversion tools distributed with the AMOS package.

#### Overlap stage

The hash-overlap program is used to compute all pair-wise alignments between the reads provided in the input.

#### Unitigger stage

The tigger module constructs a graph representation of the set of overlaps determined in the overlap stage. The overlap graph contains a node for each shotgun read, and an edge connects two nodes if the corresponding reads overlap. The unitigger then uses several reduction steps to simplify this graph, and generate a set of unitigs, based on algorithms originally developed by Myers [28,29]. Briefly, these reduction steps are:

1. **Removal of containment edges**. Reads completely contained within other reads in the input are removed from the graph.

2. **Transitive reduction**. For any set of three reads (A, B, and C), if the overlap between A and C can be inferred from the overlaps between reads A and B, and B and C, this overlap (i.e. the edge corresponding to this overlap) is removed from the graph.

3. **Unique-join collapsing**. Every simple path in the graph (paths that contain no branches, i.e. all the nodes have in- and out-degrees equal to 1) are collapsed into a single vertex. Each such vertex represents an individual unitig.

#### Consensus stage

The final stage of Minimus constructs the full multiple alignment of the reads aligned within each unitig, using as a guide the approximate placement of the reads inferred from the overlap information.

### Sequence trimming

The criteria used for trimming the vector sequence and the poor quality flanks of shotgun reads vary significantly depending on the source of the data and on the protocols employed during sequencing. In designing Minimus we, thus, opted to perform the trimming of the data with external software tools that can be customized to the specific characteristics of the data. For the examples described in this paper we followed two different approaches for sequence trimming:

1. For data where we had confidence that the Trace Archive clipping coordinates were correct (i.e. the two bacterial genomes) we simply used the coordinates provided to us.

2. For the other data-sets (zebrafish gene and mouse BACs) we followed the protocol described at [30], specifically we used the program Lucy [31] for quality trimming, followed by a k-mer based vector trimming protocol.

Note that while phrap performs some trimming based on quality values, in order to ensure consistent trimming of the data, we provided phrap with sequences already trimmed according to the protocol described above.

### Extraction of GPC3 homologues from zebrafish shotgun data

To extract the set of zebrafish shotgun reads that map to the human GPC3 gene, we built an NCBI Blast database containing the high-quality region of the zebrafish reads (obtained by removing the sequencing vector and the poor quality regions). We then aligned the protein sequence of the human GPC3 gene using tblastn with an E-value cutoff of 0.01. All reads matching GPC3 under these extremely relaxed criteria were then provided to Minimus for assembly.

## Results

To demonstrate the capabilities of Minimus we present its application to the assembly of several small data-sets: influenza A virus isolates, individual genes, and BAC clones. We compare the performance of Minimus to that of phrap [2], the "small assembler" most commonly used for such small assembly tasks. We also used Minimus to assemble two bacterial genomes, *Brucella suis*, and *Staphylococcus aureus*, to illustrate its potential use as one of the components of a complex assembly pipeline. Genome assemblers such as Atlas [9], developed at the Human Genome Sequencing Center at the Baylor College of Medicine, and Phusion [8], developed at the Sanger Center, represent such assembly pipelines. Both assemblers use a hierarchical approach to partition the reads into small sets during an initial clustering step, then assemble each of the clusters with the phrap assembler.

Before describing our results we would like to emphasize the fact that the comparisons to phrap provided below are inherently skewed due to the fact that phrap and Minimus were designed to solve different problems. These comparisons are relevant, however, because phrap has been widely applied to assembly tasks that fall outside the scope of the original intended use for this program. We will demonstrate that Minimus provides scientists with a better tool for small assembly tasks, be it the assembly of viral genomes or individual genes, or as a component in a larger assembly pipeline such as Atlas or Phusion. The high stringency of the algorithms employed by Minimus obviates the need for the complex modules commonly used (e.g., the RPphrap module of Phusion [8]) in such assembly pipelines to correct the errors introduced by phrap. In addition, the flexibility provided by Minimus' well defined interfaces and open-source license, allow scientists to adapt and extend our software as needed by their specific projects. Such enhancements are virtually impossible with phrap due to the restrictive license and code release model.

### Assembly of influenza A virus isolates

Assembling the influenza A virus is an ideal application for Minimus due to the small size of the virus. The influenza A sequencing project, currently underway at TIGR [13], has been using Minimus to assemble the genomes of more than 1400 individual isolates of the influenza virus. The sequencing pipeline at TIGR generates approximately 200 sequencing reads for each viral isolate, providing approximately 4-fold coverage of the 8 segments composing the flu genome. The assembly of the influenza genome is performed in a hierarchical manner, building a collection of contigs using Minimus with high stringency settings, then improving this assembly during two additional passes that combine Minimus with quality trimming software. In approximately 95% of the cases (J. Sitz, personal communication), this hierarchical process results in complete reconstructions of each of the segments, these data forming the substrate for genome annotation and for other subsequent analyses. The whole assembly process, including the time needed to access the database used to store the reads and the resulting assemblies, takes approximately 4 minutes. The actual time used by Minimus for assembling the data is approximately 2 seconds/segment during each of the three passes. The shotgun reads, and the assemblies produced by Minimus are made freely available to the scientific community by submission to the NCBI Trace and Assembly Archives [14].

### Assembly of individual genes

One of the applications that initially drove the development of Minimus is the assembly of an individual gene from reads "fished" out of a shotgun dataset by alignment to a homologous gene from a related organism. This application is particularly relevant to the study of large eukaryotic genomes that are being sequenced but for which no assembly has yet been made available to the scientific community. While sequencing is a highly automated process, the assembly of large genomes is a time-consuming activity that requires extensive manual intervention, particularly in the case of large, highly repetitive genomes, or genomes with highly divergent homologous chromosomes. Thus, it is not uncommon for the raw shotgun data to be deposited in the Trace Archive months, and sometimes years, before an assembly of a genome is made available, even in a draft form. This situation makes it difficult for scientists to ask questions such as "does this organism being sequenced have a homologue of gene X?", or "how many copies of gene Y are present in this genome?" Such questions are often difficult to answer even if a draft assembly is available, as evidenced, for example, by the absence of chromosome Y-linked genes in an early draft of *Drosophila pseudoobscura*; in that case, investigators found the genes of interest by directly examining the underlying shotgun data [15].

To highlight the application of Minimus to assembling individual genes directly extracted from the shotgun data, we attempted to assemble the zebrafish (*Danio rerio*) homologues to the human glypican-3 (GPC3) gene. The GPC3 gene is highly expressed during development and has been implicated in a variety of cancers as well as in the Simpson-Golabi-Behmel overgrowth syndrome (see, e.g., [16-19]). We chose this combination of organisms due to the large evolutionary distance between human and zebrafish, as well as the fluid nature of the draft assembly of the zebrafish genome (currently at version 6 and still being actively improved).

**Figure 2:**
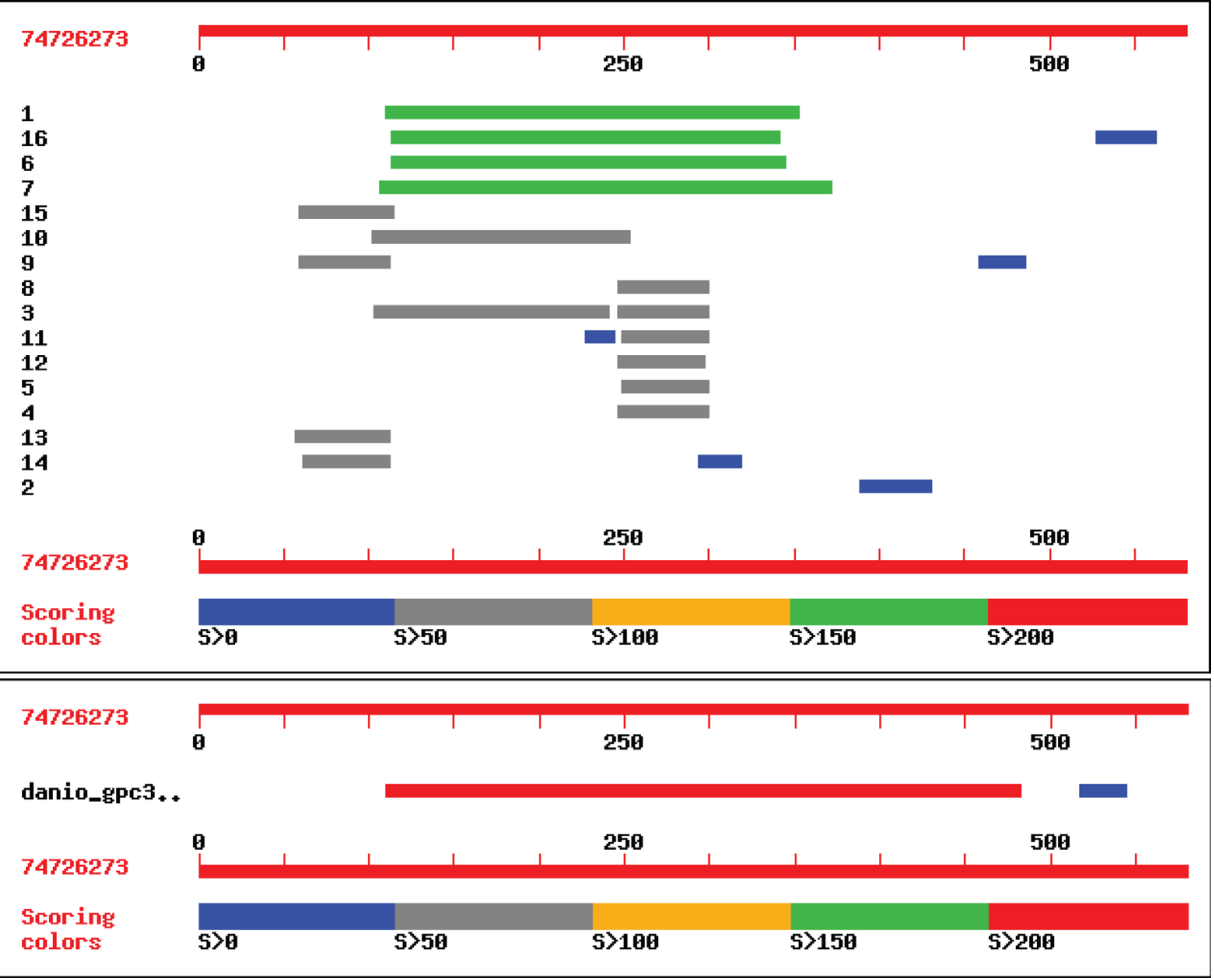
Top: Blast alignments of contigs resulting from minimus assembly of Zebrafish shotgun reads to the human GPC3 protein. The top four matches correspond to contigs that do not have any significant similarity to each other at the nucleotide level, indicating the presence of at least 4 homologues to the GPC3 gene in the Zebrafish genome. Bottom: Alignment of the Zebrafish GPC3 protein to the human GPC3 protein highlighting that the minimus assembly covers the majority of this gene.

We extracted 175 *Danio rerio *shotgun reads (from among the 24,961,699 reads publicly available at the NCBI Trace Archive) that could be mapped to the sequence of the human GPC3 protein (see Methods). We then assembled these reads using Minimus, resulting in 16 contigs, representing individual exons of the zebrafish GPC3 homologue. To ascertain the quality of the reconstruction, we mapped the individual contigs to the human gene. The overview of the alignments is shown in Figure 2 (top), indicating that approximately half of the human GPC3 gene is covered by high quality matches to four contigs generated by Minimus. Interestingly, these four contigs do not share any significant similarity at the nucleotide level, indicating the presence in zebrafish of at least four homologues to the human GPC3 gene, not unexpected given that in human GPC3 is part of a larger gene family. This result could, however, not be immediately inferred from the Zebrafish Information Network (ZFIN) – the central database for the zebrafish community. A search for "glypican" in ZFIN returns a single entry – that for the zebrafish homologue to GPC3.

To ascertain whether the incomplete coverage of the human GPC3 is due to limitations in our methodology, or to actual differences between the human and zebrafish homologues, we aligned the annotated zebrafish GPC3 homologue to the human protein (Figure 2 bottom). The alignment reveals the zebrafish GPC3 gene to be shorter than its human counterpart, consistent with our reconstruction. In fact, the Minimus contigs cover most of the zebrafish gene, with the exception of approximately 100 amino acids at the C terminus. This comparison also reveals a limitation of our approach. Short exons and/or splicing differences between the human and zebrafish homologues of the gene may prevent a simple translated search from identifying the shotgun reads necessary to reconstruct the full length gene. Despite such limitations, we believe our results show that Minimus can be successfully used as a first step in characterizing the homologues of a gene of interest in a newly sequenced organism. Furthermore, the approach we chose can be easily augmented to hierarchically recruit additional shotgun reads that extend the initial set of contigs, eventually reconstructing assemblies of entire genes. We implemented a simple version of such a procedure by also recruiting the mates for all reads identified during the translated searches. Unfortunately, the inclusion of these reads into the assembly process only resulted in marginal improvements. Better results will undoubtedly be obtained by extending this process to also incorporate reads that overlap the reconstructed contigs, however an implementation of such a procedure is beyond the scope of the current paper.

### BAC clone assembly

**Figure 3:**
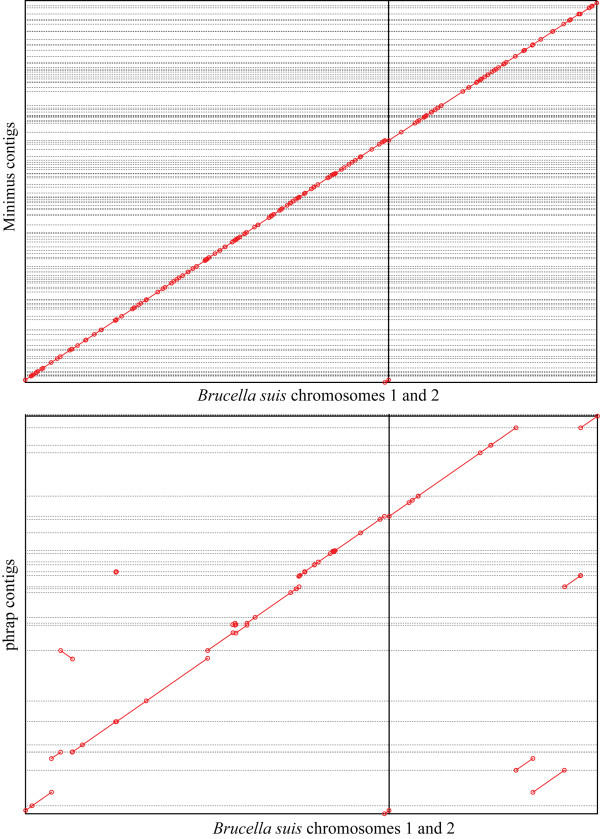
Dot plots of alignments of assemblies produced by minimus (top) and phrap (bottom) to the completed *Brucella suis *genome. The horizontal lines indicate the boundary between assembled contigs represented on the y axis. The vertical line separates between the two chromosomes of *Brucella suis *represented on the x axis. The minimus assembly (top) perfectly matches the reference sequence, as indicated by all matches lying along the main diagonal (except the contig at the bottom center, which spans the origin of the circular chromosome). The phrap assembly (bottom) shows many discrepancies with respect to the reference sequence (off-diagonal segments), including several contigs that incorrectly join segments of the two distinct chromosomes (e.g., second and third contigs from the bottom). Note that the ordering of the contigs implied by these figures is an artifact of the alignment to the reference sequence and does not correspond to the order in which the contigs were reported by the specific assembly tools. The discrepancies between the phrap assembly and the reference sequence prevent us from providing a consistent ordering for this assembly.

The sequencing of complex genomes sometimes follows a hierarchical process, whereby the DNA is first sheared into segments of between 50-150 kbp which are then amplified in *E. coli*. These segments, called Bacterial Artificial Chromosomes (BACs), are then sequenced through the shotgun method. This hierarchical approach can overcome the complexity of highly repetitive genomes (e.g., *Zea mays *[20]), and has also been applied to the exploration of environmental samples (see, e.g., [21]). The shotgun sequencing of individual BAC clones generates approximately 2000–3000 sequencing reads, which can be easily assembled with Minimus. We extracted, at random, from the NCBI Trace Archive a collection of 10 shotgun libraries generated from mouse BAC clones, and assembled these data with both Minimus and phrap. All the selected BAC clones have been finished, providing us with a "gold standard" for evaluating the correctness of the assemblies. The results of this comparison are summarized in Table 1. On these datasets, Minimus ran faster than phrap (running time was approximately 60% of the running time of phrap) and produced contigs that mapped with few errors to the finished sequences; in contrast, the phrap contigs contained up to five times as many errors as Minimus (see Figure 3 for an example of erroneous alignments to the reference sequence) when compared to each of the finished BACs. These results are unsurprising as phrap's aggressive attempts to generate longer contigs (Minimus generated contigs that were about four times smaller than phrap) often result in mis-assemblies [22]. We argue that the conservative approach taken by Minimus is preferable in the case of BAC assembly, as mis-assemblies are often difficult to identify and correct, whereas the fragmented assemblies produced by Minimus can be easily improved by utilizing mate-pair information and by using alignment information between the individual contigs.

**Table 1:** Comparison of Minimus and phrap in the assembly of 10 mouse BACs from data obtained from the NCBI Trace Archive.

BAC	BAC size (bp)	# Reads/seq. coverage		Running time	# Contigs	N50 contig size (kbp)	Coverage (%)	# errors
RP23-179K16	195,061	3685	Minimus	1 m 45 s	40	4.2	99.9	0
		8	phrap	2 m 55 s	14	16.1	99.9	2
RP23-188E5	157,996	2983	Minimus	1 m 5 s	43	4.3	99.9	0
		7	phrap	2 m 33 s	16	16.9	99.7	2
RP23-111A22	200,329	5428	Minimus	56 s	244	4.8	98.7	3
		10	phrap	1 m 43 s	183	17	98.4	14
RP23-271013	239,837	7601	Minimus	3 m 11 s	448	1.5	100.0	2
		14	phrap	6 m 30 s	329	6.3	98.6	10
RP23-283E4	178,084	5708	Minimus	3 m 49 s	713	1.4	99.9	2
		15	phrap	9 m 53 s	467	3.9	98.7	8
RP23-286D16	195,068	4969	Minimus	41 s	90	9	99.9	2
		8	phrap	1 m 22 s	264	40	99.9	5
RP23-296N18	187,242	1536	Minimus	36 s	52	6.5	99.9	0
		6	phrap	1 m 5 s	34	16.1	99.0	12
RP23-319P12	190,514	5629	Minimus	1 m 39 s	131	4.9	99.9	3
		14	phrap	2 m 29 s	139	18	98.0	18
RP23-363E23	199,409	5301	Minimus	1 m 19 s	111	5.5	99.9	3
		12	phrap	2 m 12 s	178	20	100	15
RP23-425H1	188,835	1536	Minimus	14 s	46	10	97.2	1
		6	phrap	38 s	28	21	98.4	5

Minimus ran considerably faster than phrap and produced no errors, at the expense of a larger number of contigs. Note that the table contains two quantities denoted "coverage": the sequencing coverage (reported in the #Reads/seq. coverage column) represents the total amount of DNA in the sequenced reads, divided by the size of the chromosome, i.e. the redundancy in the sequenced data; the column headed "coverage" represents the fraction of the reference sequence covered by assembled contig. The latter measure does not take into account assembly errors, i.e. partial contig matches contribute to the overall coverage.

### Assembly of bacterial genomes

In addition to the assembly of small datasets such as those described above, Minimus can also be used in conjunction with other assembly modules (such as the scaffolder Bambus [23]) to build an assembly pipeline for larger genomes. To assess the suitability of Minimus as a replacement for the phrap assembler in pipelines such as Atlas or Phusion, we compared the two assemblers on their ability to assemble two bacterial genomes: *Brucella suis*, and *Staphylococcus aureus*. Both genomes were sequenced and fully finished at TIGR, and all the sequencing reads generated for these projects are publicly available at both the NCBI Trace Archive, and from our website [24]. The availability of a finished molecule allowed us to compare the correctness of the assemblies generated by Minimus and phrap respectively, as shown in Figure 3. The results of our comparison are shown in Table 2. Similar to the case of BAC assembly, Minimus ran faster than phrap (approx. 2–5 times faster) and produced no errors. The phrap assembly contained multiple errors (8 in *B. suis *and 5 in *S. aureus*), though it produced larger contigs, 4–5 times larger than those produced by Minimus. Again, the conservative approach taken by Minimus, as well as its efficiency, make it a better choice as the core component of a genome assembly pipeline such as Atlas or Phusion. In this context, mis-assemblies may present more challenges than the relatively smaller contigs generated by Minimus.

**Table 2:** Comparison of Minimus and phrap in the assembly of two bacterial genomes (*Brucella suis *and *Staphylococcus aureus*).

Genome	Genome size (Mbp)	# Reads/seq. coverage		Running time	# Contigs	N50 contig size (kbp)	coverage (%)	# errors
*B. suis*	3.3	36080	Minimus	6 m 30 s	110	43	99.9%	0
		7.8	phrap	30 m 2 s	39	196	99.7%	8
*S. aureus*	2.9	49014	Minimus	16 m 40 s	85	51	99.8%	0
		9.2	phrap	40 m	30	190	99.7%	5

Minimus ran faster than phrap and produced no errors. However, it generated a considerably larger number of contigs. Note that the table contains two quantities denoted "coverage": the sequencing coverage (reported in the #Reads/seq. coverage column) represents the total amount of DNA in the sequenced reads, divided by the size of the chromosome, i.e. the redundancy in the sequenced data; the column headed "coverage" represents the fraction of the reference sequence covered by assembled contig. The latter measure does not take into account assembly errors, i.e. partial contig matches contribute to the overall coverage.

## Discussion

One, perhaps surprising, result of our experiments is the higher fragmentation of the BAC assemblies in comparison to the bacterial assemblies (observed both for Minimus and phrap), even though the BACs were sequenced to a deeper level of coverage. The reason for this fragmentation is the higher density of repeats in the mouse genome. Eukaryotic genomes often contain high-copy repeats that disrupt the assembly process, even within the range of a BAC insert. Such complex repeats are less frequently encountered in bacteria.

## Conclusion

We have described Minimus, a shotgun sequence assembly program designed for the assembly of small data-sets, and shown that Minimus can be successfully used to extract individual genes from shotgun data-sets, thereby providing scientists with the means to analyze newly sequenced organisms long before complete genome assemblies are made available. Due to its small size and modular design Minimus is perfectly suited to be a component of complex assembly pipelines, as shown by its use at TIGR as the main workhorse in the influenza virus sequencing pipeline. Traditionally, phrap has been used as a main component of such pipelines. We compared Minimus to phrap on two median-sized assembly tasks, BAC clones and bacterial genomes, and found that Minimus is able to perform such assemblies more efficiently and more accurately than phrap, at the cost of producing smaller contigs. We would like to emphasize the fact that it important to obtain a correct assembly, even if this assembly is fragmented. Assembly errors are often difficult to detect and correct, and are usually resolved through an expensive and time-consuming process of manual curation (no automated tools exists for this task), while fragmented assemblies can easily be improved in a high-throughput fashion by, for example, hierarchically combining the fragmented contigs based on lower-stringency overlap information. These results highlight the potential for Minimus to be used as a replacement for phrap in assembly pipelines such as Atlas or Phusion, especially as these pipelines already implement mechanisms for combining contigs. Also note that the errors in the phrap assemblies are an artifact of the greedy assembly algorithm used by phrap and cannot be resolved by simply adjusting the stringency of the assembly process.

Finally, the modular design of Minimus (and its Open Source license) allows scientists to easily fine-tune, or replace, individual components of the assembly pipeline, tailoring the execution of Minimus to the specific characteristics of the data. Such fine-tuning is impossible in phrap, partly due to its restrictive license, and also due to its monolithic design. Minimus is therefore more than a simple assembler: it can be thought of as a potential testbed for evaluating specific assembly approaches, whether for educational purposes as part of a bioinformatics curriculum, or during the conduct of research in genome assembly.

## Availability and requirements

Minimus is distributed under an Open Source license (the Artistic License) as a component of the AMOS package [25]. The details for this package are provided below.

**Project name**: AMOS

**Project homepage**: 

**Operating systems**: Unix (tested on Linux x86 and x86_64, Mac OSX, cygwin, Solaris, and Tru64)

**Programming languages**: C++, Perl

**Other requirements**: none for Minimus, some components of AMOS require the QT library

**License**: OSI Artistic License

**Any restrictions to use by non-academics**: none

Test data for running Minimus can be downloaded from the Minimus website: .

## Authors' contributions

DDS implemented the unitigger and the overall execution pipeline and ran the assemblies presented in the results section. ALD implemented the overlapper and multi-aligner programs. SLS provided the initial impetus for the design of Minimus and encouraged and oversaw the integration with the flu sequencing pipeline. MP led the design of the package, provided conversion utilities for various assembly formats, and performed the analysis of the zebrafish GPC3 homologues. All authors contributed to writing the manuscript.

## References

[B1] Sutton GG, White O, Adams MD, Kerlavage AR (1995). TIGR Assembler: A New Tool for Assembling Large Shotgun Sequencing Projects. Genome Science and Technology.

[B2] Ewing B, Green P (1998). Base-calling of automated sequencer traces using phred. II. Error probabilities. Genome Res.

[B3] Huang X, Madan A (1999). CAP3: A DNA Sequence Assembly Program. Genome Research.

[B4] Myers EW, Sutton GG, Delcher AL, Dew IM, Fasulo DP, Flanigan MJ, Kravitz SA, Mobarry CM, Reinert KH, Remington KA, Anson EL, Bolanos RA, Chou HH, Jordan CM, Halpern AL, Lonardi S, Beasley EM, Brandon RC, Chen L, Dunn PJ, Lai Z, Liang Y, Nusskern DR, Zhan M, Zhang Q, Zheng X, Rubin GM, Adams MD, Venter JC (2000). A whole-genome assembly of Drosophila. Science.

[B5] Batzoglou S, Berger B, Mesirov J, Lander ES (1999). Sequencing a Genome by Walking with Clone-End Sequences: A Mathematical Analysis. Genome Research.

[B6] Jaffe DB, Butler J, Gnerre S, Mauceli E, Lindblad-Toh K, Mesirov JP, Zody MC, Lander ES (2003). Whole-genome sequence assembly for Mammalian genomes: arachne 2. Genome Res.

[B7] Waterston RH, Lindblad-Toh K, Birney E, Rogers J, Abril JF, Agarwal P, Agarwala R, Ainscough R, Alexandersson M, An P, Antonarakis SE, Attwood J, Baertsch R, Bailey J, Barlow K, Beck S, Berry E, Birren B, Bloom T, Bork P, Botcherby M, Bray N, Brent MR, Brown DG, Brown SD, Bult C, Burton J, Butler J, Campbell RD, Carninci P, Cawley S, Chiaromonte F, Chinwalla AT, Church DM, Clamp M, Clee C, Collins FS, Cook LL, Copley RR, Coulson A, Couronne O, Cuff J, Curwen V, Cutts T, Daly M, David R, Davies J, Delehaunty KD, Deri J, Dermitzakis ET, Dewey C, Dickens NJ, Diekhans M, Dodge S, Dubchak I, Dunn DM, Eddy SR, Elnitski L, Emes RD, Eswara P, Eyras E, Felsenfeld A, Fewell GA, Flicek P, Foley K, Frankel WN, Fulton LA, Fulton RS, Furey TS, Gage D, Gibbs RA, Glusman G, Gnerre S, Goldman N, Goodstadt L, Grafham D, Graves TA, Green ED, Gregory S, Guigo R, Guyer M, Hardison RC, Haussler D, Hayashizaki Y, Hillier LW, Hinrichs A, Hlavina W, Holzer T, Hsu F, Hua A, Hubbard T, Hunt A, Jackson I, Jaffe DB, Johnson LS, Jones M, Jones TA, Joy A, Kamal M, Karlsson EK, Karolchik D, Kasprzyk A, Kawai J, Keibler E, Kells C, Kent WJ, Kirby A, Kolbe DL, Korf I, Kucherlapati RS, Kulbokas EJ, Kulp D, Landers T, Leger JP, Leonard S, Letunic I, Levine R, Li J, Li M, Lloyd C, Lucas S, Ma B, Maglott DR, Mardis ER, Matthews L, Mauceli E, Mayer JH, McCarthy M, McCombie WR, McLaren S, McLay K, McPherson JD, Meldrim J, Meredith B, Mesirov JP, Miller W, Miner TL, Mongin E, Montgomery KT, Morgan M, Mott R, Mullikin JC, Muzny DM, Nash WE, Nelson JO, Nhan MN, Nicol R, Ning Z, Nusbaum C, O'Connor MJ, Okazaki Y, Oliver K, Overton-Larty E, Pachter L, Parra G, Pepin KH, Peterson J, Pevzner P, Plumb R, Pohl CS, Poliakov A, Ponce TC, Ponting CP, Potter S, Quail M, Reymond A, Roe BA, Roskin KM, Rubin EM, Rust AG, Santos R, Sapojnikov V, Schultz B, Schultz J, Schwartz MS, Schwartz S, Scott C, Seaman S, Searle S, Sharpe T, Sheridan A, Shownkeen R, Sims S, Singer JB, Slater G, Smit A, Smith DR, Spencer B, Stabenau A, Stange-Thomann N, Sugnet C, Suyama M, Tesler G, Thompson J, Torrents D, Trevaskis E, Tromp J, Ucla C, Ureta-Vidal A, Vinson JP, Von Niederhausern AC, Wade CM, Wall M, Weber RJ, Weiss RB, Wendl MC, West AP, Wetterstrand K, Wheeler R, Whelan S, Wierzbowski J, Willey D, Williams S, Wilson RK, Winter E, Worley KC, Wyman D, Yang S, Yang SP, Zdobnov EM, Zody MC, Lander ES (2002). Initial sequencing and comparative analysis of the mouse genome. Nature.

[B8] Mullikin JC, Ning Z (2003). The phusion assembler. Genome Res.

[B9] Havlak P, Chen R, Durbin KJ, Egan A, Ren Y, Song XZ, Weinstock GM, Gibbs RA (2004). The Atlas genome assembly system. Genome Res.

[B10] Aparicio S, Chapman J, Stupka E, Putnam N, Chia JM, Dehal P, Christoffels A, Rash S, Hoon S, Smit A, Gelpke MD, Roach J, Oh T, Ho IY, Wong M, Detter C, Verhoef F, Predki P, Tay A, Lucas S, Richardson P, Smith SF, Clark MS, Edwards YJ, Doggett N, Zharkikh A, Tavtigian SV, Pruss D, Barnstead M, Evans C, Baden H, Powell J, Glusman G, Rowen L, Hood L, Tan YH, Elgar G, Hawkins T, Venkatesh B, Rokhsar D, Brenner S (2002). Whole-genome shotgun assembly and analysis of the genome of Fugu rubripes. Science.

[B11] Fleischmann RD, Adams MD, White O, Clayton RA, Kirkness EF, Kerlavage AR, Bult CJ, Tomb JF, Dougherty BA, Merrick JM (1995). Whole-genome random sequencing and assembly of Haemophilus influenzae Rd. Science.

[B12] NCBI Trace Archive. http://www.ncbi.nlm.nih.gov/Traces.

[B13] Ghedin E, Sengamalay NA, Shumway M, Zaborsky J, Feldblyum T, Subbu V, Spiro DJ, Sitz J, Koo H, Bolotov P, Dernovoy D, Tatusova T, Bao Y, St George K, Taylor J, Lipman DJ, Fraser CM, Taubenberger JK, Salzberg SL (2005). Large-scale sequencing of human influenza reveals the dynamic nature of viral genome evolution. Nature.

[B14] Salzberg SL, Church D, DiCuccio M, Yaschenko E, Ostell J (2004). The genome Assembly Archive: a new public resource. PLoS Biol.

[B15] Carvalho AB, Clark AG (2005). Y chromosome of D. pseudoobscura is not homologous to the ancestral Drosophila Y. Science.

[B16] Blackhall FH, Merry CL, Davies EJ, Jayson GC (2001). Heparan sulfate proteoglycans and cancer. Br J Cancer.

[B17] Pilia G, Hughes-Benzie RM, MacKenzie A, Baybayan P, Chen EY, Huber R, Neri G, Cao A, Forabosco A, Schlessinger D (1996). Mutations in GPC3, a glypican gene, cause the Simpson-Golabi-Behmel overgrowth syndrome. Nat Genet.

[B18] Toretsky JA, Zitomersky NL, Eskenazi AE, Voigt RW, Strauch ED, Sun CC, Huber R, Meltzer SJ, Schlessinger D (2001). Glypican-3 expression in Wilms tumor and hepatoblastoma. J Pediatr Hematol Oncol.

[B19] Xiang YY, Ladeda V, Filmus J (2001). Glypican-3 expression is silenced in human breast cancer. Oncogene.

[B20] MaizeGDB - Maize Genetics and Genomics Database. http://www.maizegdb.org.

[B21] Beja O, Aravind L, Koonin EV, Suzuki MT, Hadd A, Nguyen LP, Jovanovich SB, Gates CM, Feldman RA, Spudich JL, Spudich EN, DeLong EF (2000). Bacterial rhodopsin: evidence for a new type of phototrophy in the sea. Science.

[B22] Pop M, Salzberg SL, Shumway M (2002). Genome sequence assembly: Algorithms and issues. Computer.

[B23] Pop M, Kosack DS, Salzberg SL (2004). Hierarchical scaffolding with Bambus. Genome Res.

[B24] Benchmark Data for Genome Assembly. http://www.cbcb.umd.edu/research/benchmark.shtml.

[B25] AMOS - A Modular Open-Source Assembler. http://amos.sourceforge.net.

[B26] Peltola H, Soderlund H, Ukkonen E (1984). SEQAID: a DNA sequence assembling program based on a mathematical model. Nucleic Acids Res.

[B27] Roberts M, Hayes W, Hunt BR, Mount SM, Yorke JA (2004). Reducing storage requirements for biological sequence comparison. Bioinformatics.

[B28] Myers EW (1995). Toward Simplifying and Accurately Formulating Fragment Assembly. J Comp Bio.

[B29] Myers EW (2005). The fragment assembly string graph. Bioinformatics.

[B30] Running Celera Assembler: Trimming the input data. http://www.cbcb.umd.edu/research/CeleraAssembler.shtml#trimmingtheinput.

[B31] Chou HH, Holmes MH (2001). DNA sequence quality trimming and vector removal. Bioinformatics.

